# Investigations into genome diversity of *Haemophilus influenzae* using whole genome sequencing of clinical isolates and laboratory transformants

**DOI:** 10.1186/1471-2180-12-273

**Published:** 2012-11-23

**Authors:** Peter M Power, Stephen D Bentley, Julian Parkhill, E Richard Moxon, Derek W Hood

**Affiliations:** 1 University of Oxford Department of Paediatrics, Medical Sciences Division, John Radcliffe Hospital, Headington, Oxford OX3 9DU, UK; 2 Pathogen Genomics, Wellcome Trust Sanger Institute, Wellcome Trust Genome Campus, Hinxton, Cambridge, CB10 1SA, UK; 3 Nuffield Department of Clinical Medicine, University of Oxford, John Radcliffe Hospital, Headington, Oxford, OX3 9DU, UK

**Keywords:** *Haemophilus influenzae*, Genome sequence, Population structure, Transformation

## Abstract

**Background:**

*Haemophilus influenzae* is an important human commensal pathogen associated with significant levels of disease. High-throughput DNA sequencing was used to investigate differences in genome content within this species.

**Results:**

Genomic DNA sequence was obtained from 85 strains of *H. influenzae* and from other related species, selected based on geographical site of isolation, disease association and documented genotypic and phenotypic differences. When compared by Mauve alignment these indicated groupings of *H. influenzae* that were consistent with previously published analyses**; c**apsule expressing strains fell into two distinct groups and those of serotype b (Hib) were found in two closely positioned lineages. For 18 Hib strains representing both lineages we found many discrete regions (up to 40% of the total genome) displaying sequence variation when compared to a common reference strain. Evidence that this naturally occurring pattern of inter-strain variation in *H. influenzae* can be mediated by transformation was obtained through sequencing DNA obtained from a pool of 200 independent transformants of a recipient (strain Rd) using donor DNA from a heterologous Hib strain (Eagan).

**Conclusion:**

Much of the inter-strain variation in genome sequence in *H. influenzae* is likely the result of inter-strain exchanges of DNA, most plausibly through transformation.

## Background

*Haemophilus influenzae* is a frequently isolated member of the commensal microbiota of the human nasopharynx that also causes a variety of diseases including invasive infections (meningitis and septicaemia) as well as diseases resulting from contiguous spread within the respiratory tract, such as otitis media, pneumonia, conjunctivitis, epiglottitis, and exacerbations of chronic obstructive pulmonary disease (COPD). An important question is the extent to which genotypic variation within the species, especially that which affects surface expressed structures such as capsule, lipopolysaccharide (LPS) and outer membrane proteins (OMPs), influences pathogenesis.

Within naturally occurring populations of transformable bacteria, it has been proposed that each strain in a population contributes to and can acquire genes from the pan-genome (the superset of all genes of the species) [1-3]. This hypothesis suggests that genetic exchange, especially through transformation-mediated homologous recombination, plays a major role in shaping the diversity of *H. influenzae,* and that these variations affect commensal and virulence behaviour. If so, investigations that detail the extent of the genomic diversity of the species and the mechanisms by which this diversity is transferred between strains are important for understanding both the population dynamics and characterising the genetic basis of the differences in severity and spectrum of disease associated with particular strains.

*H. influenzae* was the first free-living organism to have its genome sequenced [4]. This breakthrough marked the beginning of the “genome age” and offered the potential to gain more detailed information on the population structure of the species than current methods, including multi-locus sequence typing (MLST) [5-13]. However, the availability of complete genome sequences for only a few strains is insufficient to interrogate the extent of the genetic diversity of *H. influenzae* and its close species relatives. In this study, a detailed analysis of 18 *H. influenzae* type b (Hib) strains compared to a common reference identified regions of high SNP density or sequence mismatches consistent with inter-strain exchange of DNA most plausibly derived from other *H. influenzae* strains through transformation, rather than phage or conjugative transfer. Further evidence for the role of transformation in the import of novel sequence flanked by regions of DNA found in both the donor and recipient was obtained through sequencing DNA obtained from a pool of strains each transformed with DNA from a heterologous donor Hib strain.

## Results

### Whole genome sequencing of 85 strains of *Haemophilus* spp

The genomes of 96 strains of *Haemophilus spp.* (Table 1) were sequenced using the Illumina GAII platform. For 85 of these strains where sufficient coverage had been attained, genome sequences of between 1.27 Mbp to 1.91 Mbp in length were assembled by Velvet [14] (Table 1). The sequencing and assembly resulted in between 351 and 1521 contigs per strain with a median of 785 contigs per assembled genome. The genome sequences were partial and the %G+C content of these (37.94 to 40.39%) was higher than expected based on data from other completed *H. influenzae* genomes (38.01-38.15%). DNA similarity searches and mapping of the sequence reads using MAQ [15] confirmed that the higher %G+C regions of the genomes had been preferentially sequenced, a known issue with early versions of the Illumina sequencing chemistry. We estimated the average genome coverage to be 83%, based on comparison with extant complete *H. influenzae* genome sequences; this data represents a ten-fold increase in the amount of genome sequence information available for *H. influenzae.*

**Table 1:** ***Haemophilus *strains selected for study**

**Strain name**	**Type**	**Geographic location**	**Year**	**Length of sequence (Mb)**	**Disease/ Site of isolation**
RM7190	a	Malaysia	1973	1.5	meningitis
RM6062	a	England	1965	1.5	nasopharynx
RM6064	a	England	1966	1.5	pleural fluid
RM6073	a	England	1966	1.6	bronchitis
RM7017	b	Ghana	1983	1.6	CSF
RM7060	b	New York, USA	1971	1.5	nasopharynx
RM7414	b	Kenya	1980’s	1.5	
RM7419	b	Kenya	1980’s	1.5	
RM7651	b	Norway	1976	1.7	
DC11238	b	UK	2003	1.8	meningitis
DC800	b	UK	1989	1.9	meningitis
DC8708	b	UK	2000	1.8	
DCG1574	b	Gambia	1993	1.8	nasopharynx
Eagan	b			1.5	
RM7578	b	Switzerland	1983	1.8	
RM7582	b	RSA	1980’s	1.8	
RM7598	b	USA	1985	1.8	
RM7018	b*	Ghana	1983	1.4	CSF
RM7122	b*	Australia	<1984	1.5	meningitis
RM7459	b*	Iceland	1984	1.4	CSF
RM7465	b*	Iceland	1985	1.6	CSF
RM7617	b*	Malaysia	1970’s	1.5	CSF
RM6132	c	England	1964	1.6	chronic sinusitis
RM6134	c	England	1975	1.4	abscess
RM7422	c	Kenya	1986	1.4	
RM6158	e	England	1962	1.7	cystic fibrosis
RM6237	f	England	1963	1.4	nasal discharge
RM7283	f	Malaysia	1972	1.5	trachea
RM7290	f	Malaysia	1974	1.5	trachea(malnutrition)
PLMIOG2822H-L	*H. haemolyticus*			1.6	
PLh.hlnctc10659T	*H. haemolyticus*			1.6	
PLHparaphorH-L	*H. paraphrophilus*			1.7	
PLMIOG2838H-L	*H. haemolyticus*			1.4	
DCMO-099-5-LST-8	*H. parainfluenzae*	UK	1997	1.7	nasopharynx (commensal)
DCMO-099-8-MST-8	*H. parainfluenzae*	UK	1997	1.6	nasopharynx (commensal)
DCO-CFE24-1-T2ST-27	*H. parainfluenzae*	UK	2001	1.8	nasopharynx (commensal)
DCO-OM30-1-A1	*H. parainfluenzae*	UK	2001	1.6	nasopharynx (commensal)
DCT2T1ST-34	*H. parainfluenzae*	Gambia	2001	1.9	nasopharynx (commensal)
DCT5A1ST-41	*H. parainfluenzae*	Gambia	2001	1.9	nasopharynx (commensal)
DCT7B2ST-47	*H. parainfluenzae*	Gambia	2001	1.8	nasopharynx (commensal)
DCT8A1ST-52	*H. parainfluenzae*	Gambia	2001	1.9	nasopharynx (commensal)
RY15	*H. parainfluenzae*			1.7	nasopharynx (commensal)
RY20	*H. parainfluenzae*			1.7	nasopharynx (commensal)
RY22	*H. parainfluenzae*			1.9	nasopharynx (commensal)
RY8	*H. parainfluenzae*			1.7	nasopharynx (commensal)
DCT2B3ST-33	hybrid	Gambia	2001	1.4	nasopharynx (commensal)
DCG-T53T1	hybrid	Gambia	2001	1.5	nasopharynx (commensal)
DCT8B3ST-51	hybrid	Gambia	2001	1.5	nasopharynx (commensal)
DH1500spain	NTHi	Spain	2000	1.4	COPD
DH1559spain	NTHi	Spain	2000	1.5	COPD
DH1630spain	NTHi	Spain	2000	1.3	COPD
DH398spain	NTHi	Spain	2000	1.5	COPD
Fi176	NTHi	Finland	1995	1.5	otitis media
Fi723	NTHi	Finland	1995	1.6	otitis media
Fi981	NTHi	Finland	1995	1.7	otitis media
RM6011	NTHi	UK	1984	1.3	meningitis
RM6019	NTHi	UK	1984	1.3	meningitis
RM6033	NTHi	UK	1984	1.5	pus hydrosalpinx
RM6051	NTHi	UK	1985	1.5	CSF
RM7028	NTHi	PNG	1980’s	1.5	blood
RM7308	NTHi	South Korea	1984	1.5	nasopharynx
RM7309	NTHi	South Korea	1984	1.5	nasopharynx
RM7347	NTHi	USA	1985	1.4	sputum
RM7448	NTHi	Iceland	1978	1.4	blood
RM7477	NTHi	Iceland	1986	1.6	
RM7490	NTHi	RSA	1980’s	1.6	CSF
DH1513spain	NTHi	Spain	2000	1.5	COPD
Fi1180	NTHi	Finland	1995	1.6	otitis media
Fi162	NTHi	Finland	1995	1.7	otitis media
Fi667	NTHi	Finland	1995	1.7	otitis media
RM7029	NTHi	PNG	1980’s	1.6	blood
RM7637	NTHi	China	1971	1.4	sputum
DC7331	NTHi	UK	1997	1.8	meningitis
DC7654	NTHi	UK	1997	1.8	blood
DC7695	NTHi	UK	1997	1.9	CSF
DCg2120	NTHi	Gambia		1.8	nasopharynx
DCH3151	NTHi	Gambia	1993	1.8	pneumonia
DCO-OM33-2B3ST-21	NTHi	UK	2001	1.5	nasopharynx
PLMIOG2819				1.5	
PLMIOG2820				1.5	
RM6006				1.4	
PLMIOG2836				1.7	
DCMO-009-14-S-TR-ST-12		UK	1998	1.6	nasopharynx
PL10839T				1.6	
PLMIOG2837				1.6	
RM7054	NTHi	USA	1984		blood (sepsis)
Fi1247	NTHi	Finland	1995		otitis media
Fi1124	NTHi	Finland	1995		otitis media
Fi486	NTHi	Finland	1995		otitis media
Fi432	NTHi	Finland	1995		otitis media
RM7068	NTHi	PNG			pneumonia
Fi285	NTHi	Finland	1995		otitis media
PP H.parasuis	NTHi				
RM7876					
Fi1200	NTHi	Finland	1995		otitis media
RM7066					

The 96 strains selected for genome sequencing are listed along with respective information on serotype, isolation, and associated disease. The total length of the genome sequence following assembly is listed (to the nearest 0.1 Mbp) for each strain. The 11 strains below the horizontal line are those for which the quality of the assembled genome sequence was insufficient for the sequence data to be included in subsequent analyses.

* Strains were originally designated as NT.

**Figure 1:**
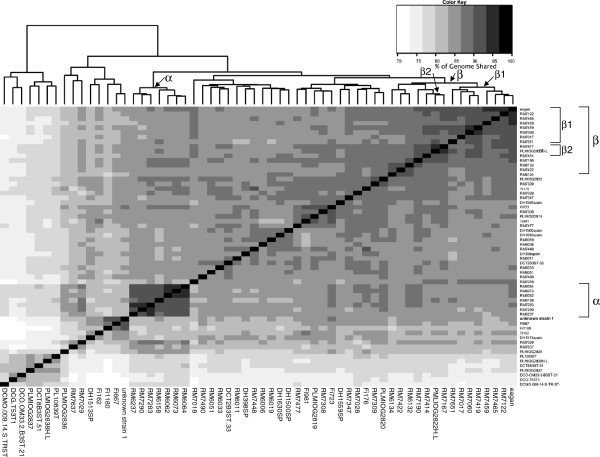
**Whole genome heat map, constructed by Mauve, to achieve pairwise percentage of genome sequence alignment.** Pair-wise Mauve alignments were conducted with 60 *H. influenzae* and *H. haemolyticus* genome sequences from strains included on a single sequencing flow cell. For each pair-wise comparison the length of the alignment achieved, expressed as the percentage of the total sequence length, was calculated and a distance matrix created. The heat map was created using the R statistical package and shows the clustered genomes determined by the default R heatmap function clustering methods ( http://www.r-project.org/). At the top of the figure, an indication of the relatedness between genomes is given. Mauve achieved pairwise genome sequence alignments of between 69.8 and 94.4% across our range of genomes. Strains are listed in the same order on the x and y axes; groupings discussed in the text are indicated along the top axis and the relevant strains are indicated by brackets on the right hand side axis, labelled with a Greek letter.

The genome assemblies were aligned in a pair-wise fashion using Mauve [16]. The length of the aligned portion of genomes achieved between any pair of strains, expressed as a percentage of the genome sequence length, was used as a measure of the relatedness of the strains. These pair-wise relationships were displayed as a heatmap using the R statistical package included within the analysis software (Figure 1). This method of ordering of strains is dependent on each having a similar degree of sequence coverage, and hence assembly length, thus the analysis was confined to data for the 60 genomes of *H. influenzae* and *H. haemolyticus* sequenced in the same flow cell (see Methods). A tree obtained following a simpler SNP-based analysis of the genome sequences (Additional file 1: Figure S1) gave an overall similar grouping of strains, validating the output from the Mauve analysis.

### Whole genome alignment reveals details of the genetic relationships of *H. influenzae* type b strains

Although this approach cannot give information on detailed phylogenetic relationships, it did allow the identification of some major groups and many sub-groups of strains (Figure 1) that were plausible and consistent with previously published analyses. Strains expressing a capsule fell into two groups (α and β in Figure 1) distinct from other *H. influenzae* strains. Hib strains were found only in two closely positioned sub-groups (β1 and β2 in Figure 1) which, interestingly, also included four strains that had originally been designated as non-typeable by serological tests (Table 1). BLAST analysis of these four genome sequences revealed a type b capsule locus in each case and all four strains were recorded as being isolated from CSF, or were associated with meningitis. We suppose that loss, or reduction, of type b capsule expression in these strains may have occurred during isolation and/or culture in the laboratory.

**Figure 2:**
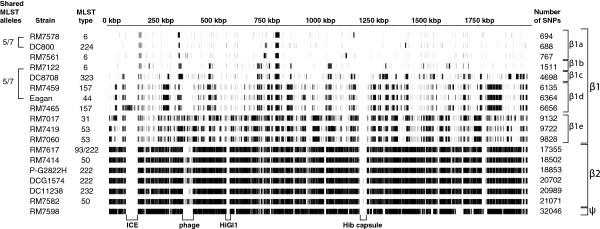
**SNPs of *H. influenzae *type b strain sequences when compared with Hib strain 10810.** The complete genome sequence of the Hib strain 10810 was used as a reference against which the sequence reads of each strain were mapped using MAQ. Each vertical black line represents the location of a SNP. The equivalent groupings to those identified in Figure 1 are labelled on the right hand side. Regions marked at the bottom of the figure represent genome segments which are present in the reference strain 10810 but that may not be found in all other strains. The brackets on the left hand side of the figure indicate the number of MLST alleles shared between the pairs of genomes indicated; the sequence type (ST) of each strain is indicated to the right of its name.

Based on the output from Mauve analysis, we selected Hib strains to analyse, in more depth, the differences in genome content that shape this level of diversity within the species. We used read-mapping by MAQ to investigate single nucleotide polymorphisms (SNPs) between 18 Hib strains included in our genome sequence database and a common reference (Table 1, Figure 2). Strain RM7018, originally designated non-typeable was excluded as it was not a member of this Hib group based on Mauve analysis (Figure 1). Conversely, we included strain PLMIOG2822H-L, a type b strain that had been wrongly classified as *H. haemolyticus*. Sequence reads were mapped onto a complete reference Hib genome sequence (strain 10810; Genbank FQ312006.1) and used to identify SNPs for all Hib strains. The Hib groupings observed (Figure 2) were essentially the same as those observed by Mauve analysis (Figure 1). Based on the location and number of SNPs, the β1 strains can be sub-grouped into β1a-β1e, and strain RM7598 contains sufficient differences to constitute a separate group (ψ) from the β2 strains (Figure 2). Genome sequence data provides greater resolution in characterising divergence of strains that share identical or similar MLST profiles. For example, when we compared the patterns of SNPs of the sub-grouped β1a-β1e strains to their respective MLSTs, we found that strains RM7578 and DC800 shared similar blocks of SNPs when compared to strain 10810, in a pattern indicative of a common vertical inheritance. Strains RM7578 and DC800 had differed by two MLST alleles (Figure 2). Strains RM7122 and Eagan also differed by two MLST alleles but differed by 4,853 SNPs in comparison to strain 10810.

### Distribution of SNPs in Hib strains indicates putative transformation events and evidence of loss and gain of genes between Hib strains

The SNPs in the genomes representative of the three lineages of Hib strains (β1, β2 and ψ) were compared in more detail to the Hib strain 10810 (a β1 strain) reference genome (Figure 2). With respect to the reference genome, β1 strains had between 688 and 9,828 SNPs and β2 strains had between 17,355 and 21,071 SNPs (Figure 2). In the β1 strains the number of SNP-dense regions was low, whereas there were many more SNPs in the β2 strains due to their greater phylogenetic difference from the reference. The single ψ strain had 32,828 SNPs (not shown in Figure 2). Although the β2 strains and the ψ strain had a broadly similar number of SNPs, they were clustered in patterns that were distinct between the groups, a finding consistent with regions of high SNP density likely representing distinct recombination events.

**Figure 3:**
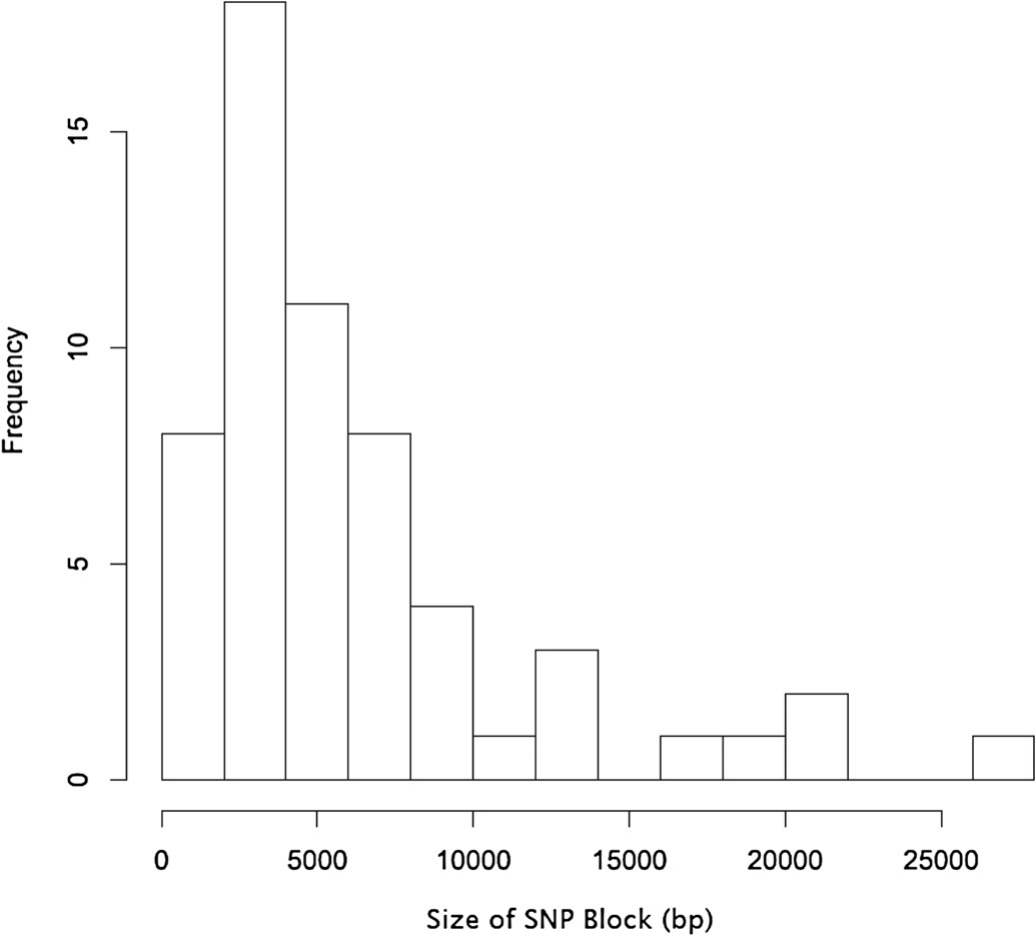
**Size of SNP blocks found in the β1 group of Hib strains.** This histogram represents the frequency of different sizes of SNP blocks (as defined in the text) in the genomes of β1 *H. influenzae* type b strains.

We hypothesised that “blocks” of DNA sequence with a high frequency of SNPs, separated by regions of the genome with low SNP density, could each represent an individual transformation event (Figure 2). To investigate this, we analysed two strains (RM7578 and RM7122) that have the same multi-locus sequence type. RM7578, the strain most closely related to the reference strain 10810, has five blocks of SNPs. For this analysis, blocks were defined as contiguous regions containing at least 30 SNPs, with each SNP separated by no more than 300 bp. 91% of 694 SNPs between strains RM7578 and 10810 were found within these five blocks, amounting in total to 23.5 kbp of DNA, or 1.2% of the genome. Strain RM7122 had 15 blocks of SNPs when compared to strain 10810, equivalent to 2.4% of the genome. In the β1 strains, the size of these blocks ranged from less than 0.5 to more than 25 kbp, with a median size of 4.8 kbp (Figure 3), findings within the range recently reported experimentally for *H. influenzae* strains [17]. We concluded that the blocks of SNPs identified between the closely related Hib strains represented recombination events, resulting in allelic exchanges that could delete or insert novel DNA, including whole genes.

Inserted or deleted regions of DNA in Hib strains, relative to the genome sequence of reference strain 10810, were identified by BLASTN searches and the ACT genome browser. For a closely related strain, DC800, an example of insertion of a novel block of SNPs, mediated through transfer of DNA from an unknown donor, was identified. This inserted DNA included a putative gene flanked by regions of significant similarity. As a further example, comparison between two more divergent genomes (RM7060 and 10810) revealed at least 16 regions of DNA, each over 500 bp in length, that were present in one strain but not the other (Table 2). These regions constitute over 17.1 kbp or approximately 1% of the genome sequence length. Similarity searches using BLASTX revealed that eleven of the 16 regions contained sequences associated with phage proteins found in *H. influenzae* and related species. The remaining five regions encoded a putative tRNA-dihydrouridine synthase C, a predicted transcriptional regulator (NikR), a transport protein, and Hia and Hap proteins.

**Table 2:** **Regions in the *H. influenzae *strain RM7060 genome not found in strain 10810**

**Accession number**	**Highest match by BLASTX analysis**	**Species**
ZP_01791522	NikR predicted transcriptional regulator	*H. influenzae* PittAA
AAL79955	Hia/YadA-like similar to neisserial GNA992	*H. influenzae* nontypeable strain 1860A
AAM74927	Hap peptidase S6	*H. influenzae* HK274
ZP_05977792	putative carboxylate/amino acid/amine transporter	*Neisseria mucosa*
P46495	Putative integrase/recombinase HI_1572	*H. influenzae*
ZP_00134779	Phage-related protein, tail component	*Actinobacillus pleuropneumoniae*
YP_001968298	Phage-related protein, tail component	*Actinobacillus pleuropneumoniae*
ZP_01791539	Mu-like prophage protein	*H. influenzae* PittAA
YP_003007008	Phage-related minor tail protein	*Aggregatibacter aphrophilus* NJ8700
ZP_01791533	putative phage tail component	*H. influenzae* PittAA
YP_001290203.1	tRNA-dihydrouridine synthase C	*H. influenzae* PittEE
YP_001053216.1	predicted bacteriophage tail assembly protein	*Actinobacillus pleuropneumoniae* L20
ZP_05990265	hypothetical protein COK_2151	*Mannheimia haemolytica*
ZP_04753126	possible prophage antirepressor	*Actinobacillus minor* NM305
ZP_04464399	Phage Mu protein F like protein	*H. influenzae* 6P18H1
YP_003007004	phage protein	*Aggregatibacter aphrophilus*

### Experimental assessment of *H. influenzae* transformation

**Figure 4:**
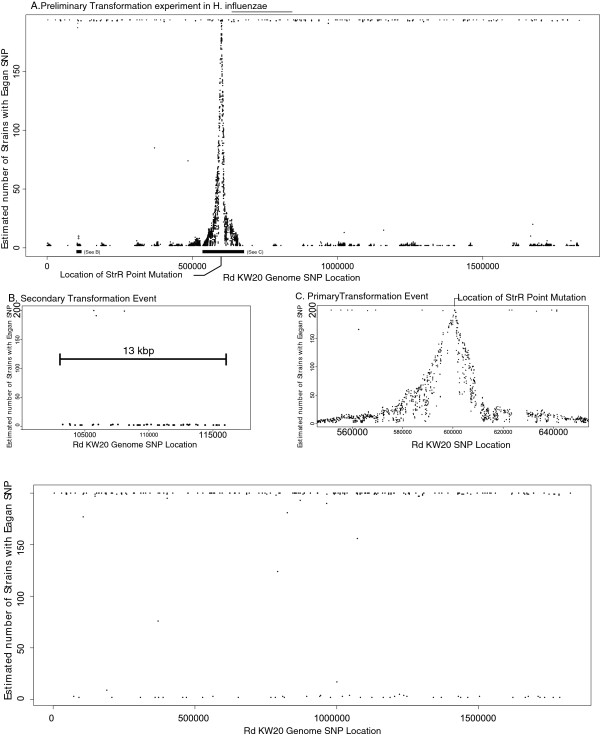
**Frequency of Eaganstr**^**R**^**and Eagan SNPs in the Rd+Eaganstr**^**R**^**and Rd+Eagan transformants.** Panel **A**; Location and frequency of Eaganstr^R^ specific SNPs plotted as estimated number of strains (y-axis) against location in RdKW20 genome sequence (x-axis) using SNPSeeker. MAQ was used to identify SNPs in the pooled sequences from 200 transformants. The location of the str^R^ point mutation is indicated. Panel **B**; A magnified view of one region marked on Panel A showing a putative secondary transformation event. The extent of the chromosomal region involved with this predicted transformation event (13 kbp) is marked. Panel **C**; A magnified view of the primary transformation event from Panel A with the location of the str^R^ point mutation marked. Panel **D**; The location and frequency of Eagan-specific SNPs in the genome of pooled Rd+Eagan transformants (200); Eagan unmarked (wild-type) genomic DNA was used as the donor.

High throughput sequencing provides a useful experimental tool to examine in detail the recombination events associated with the transfer and exchange of DNA between *H. influenzae* strains through transformation. To this end, we investigated the transformation of DNA from a Hib strain donor into a high efficiency recipient strain. To ensure that each transformant was the result of a recombination event we used a spontaneous, high level streptomycin resistant (str^R^) derivative of strain Eagan (Eaganstr^R^), possessing a point mutation in *rpoB*. Spontaneous str^R^ mutants were infrequent (<10^-10^ in control transformations of Rd using streptomycin-sensitive Eagan DNA). Compared to strain Rd, the donor strain Eagan genome sequence had 18,789 SNPs relatively uniformly distributed throughout the genome (an average density of 10.3 SNPs per kbp) including the region around *rpoB*, the location of the str^R^ mutation. Following transformation and selection on streptomycin, 200 independent Rd+Eaganstr^R^ colonies were pooled, the genomic DNA sequenced and mapped to the Rd reference genome sequence using the MAQ programme to identify SNPs. The number of Rd+Eaganstr^R^ transformants carrying each SNP was estimated from the pooled sequence using the SNPSeeker script [18] and is plotted in Figure 4. 4,501 SNPs consistent with transfer from Eagan (i.e. they were in the same genome location as the Eagan SNPs identified above) were found in the Rd+Eaganstr^R^ transformants. We identified 202 SNPs that were common to all respective sequence reads, were not linked closely to other SNPs and were found in both Rd+Eaganstr^R^ and Rd+Eagan transformants obtained in control experiments using non-str^R^ Eagan DNA as donor. We conclude that these SNPs were consistent with, and most likely explained by, errors within the reported Rd genome sequence published in 1995. Another possibility, not mutually exclusive with sequencing errors, could be sequence drift in our laboratory strain (RM118) when compared to the sequenced isolate (Rd KW20). This level of error is similar to the several hundred SNPs reported upon re-sequencing of strain Rd by other investigators [17] and comparable with the 243 discrepancies found between the original 1997 *E. coli* strain MG1655 genome sequence [19] and the 2006 re-sequencing [20] of the same strain.

In the Rd+Eaganstr^R^ transformants, a large peak in SNP density centred on the site of the point mutation in *rpoB* conferring str^R^ (Figure 4). Moving outwards from this central SNP peak, the Eagan-specific SNPs decrease at a relatively constant rate such that within 10 kbp of the str^R^ mutation the frequency of strains containing Eagan-specific SNPs decreases at approximately 1 strain/100 bp. Across the 200 transformants, the region of the genome involved in recombination events centred on the str^R^ locus would appear to span an arc of the genome over 80 kbp in size (Figure 4). Given that the str^R^ locus can be at any location in the recombined block of DNA, this indicates a maximum size for the recombined block of at least 40 kbp.

In addition to the intense peak centred on the str^R^ conferring SNP, secondary small peaks of SNPs can be observed at other locations in the genome. These secondary peaks contain Eagan strain-specific SNPs at a frequency of approximately 0.5% (Figure 4), a finding consistent with the occurrence of secondary transformation events in individual strains. These data serve to emphasize the significant impact of transformation in promoting changes in genome sequence between strains through the frequent uptake and recombination of one or more fragments of chromosomal DNA.

## Discussion

The sequencing of whole genomes from multiple strains provides a powerful means by which to examine the diversity within a bacterial species. We sequenced the genomes of 96 selected strains of *H. influenzae* and closely related *Haemophilus* spp. The approximately 25 times depth of coverage for the genomes provides a substantial increase in the existing sequence information that can expand our understanding of the gene content and organisation of *H. influenzae.* The potential role of horizontal transfer of DNA through transformation in shaping the diversity of *H. influenzae* is illustrated by our detailed analysis of SNPs in the genome sequences obtained for 18 *H. influenzae* type b (Hib) strains. Through pair-wise alignment of genome sequences, we identified regions of high SNP density (range between 3 to 40.5% of genome length), or sequence mismatches, that were consistent with inter-strain exchange of DNA. Further, in the six strains most closely related to the reference genome of strain 10810, we identified the beginnings and ends of these “blocks” that were up to 25 kbp in size with a median size of 4.8 kbp (approx. 1.5% and 0.3% of the entire genome respectively). Strains of identical MLST type display allelic variation, insertions and deletions that can include complete genes most plausibly derived from other *H. influenzae* strains through transformation. These variations may be associated with important biological differences since they can involve sequences within genes such as *hap* and *hif* that are determinants of microbial-host interaction.

In a recent publication (17), Mell and colleagues allude to the natural variation within *H. influenzae* but do not characterise it. Here we document both the details and pattern of such sequence variation in several Hib strains, variations that are consistent with recombination, most plausibly achieved through DNA transformation. To provide further independent evidence for the role of transformation, we analysed 200 laboratory transformants that were made using donor and recipient strains of known genotypes.

Each transformant contained clusters of donor-specific SNPs that represent recombinational events through transformation. The sizes of the respective chromosomal segments involved are evidently up to 40 kbp in some transformants, somewhat larger than those reported recently (8.1 ± 4.5 kbp) for other transformations carried out in *H. influenzae*[17]. Both the extent of the region of the chromosome involved in recombination events, and the possibility of secondary transformation events targeting other positions in the genome, provide good evidence indicating the potential for transformation to substantially impact on genome evolution in this bacterium. Our findings provide evidence that transformation-mediated homologous recombination plays a major role in shaping the diversity of natural *H. influenzae* populations and that individual strains contribute to and can acquire genes from the superset of all genes of the species [1-3] as has been shown also in other bacteria such as *Streptococcus pneumoniae*[21]. The “pan genome” is a resource from which specific strains can draw to allow the effective trialling of new alleles and genes in different genome backgrounds and which, through natural selection, promote survival and adaptation of *H. influenzae* within its obligate host, humans. The significant genetic divergence of genomic sequence, documented here for type b strains, but doubtless characteristic of the species as a whole, can provide information about the biological differences between strains that may determine in part the variations in commensal and pathogenic behaviour of the species.

The availability of whole genome sequencing raises the question of how best to determine the relatedness of strains of bacteria, especially in species where there is known to be substantial recombination. For *H. influenzae,* the relationships between strains inferred by the number of shared genes and the sequence similarity in house-keeping genes yield different tree topologies [3], indicating that the assumptions which underlie these methods do not reconcile phylogenetic relationships. Transformation and other mechanisms of recombination in *H. influenzae* are strong forces which can distort the perceived phylogenetic relationships between strains based on sequence similarity. It is evident from the strains examined in detail in this study that despite the genetic variation identified, there is considerable conservation of the genome between most strains. However, there are genetic elements in *H. influenzae* genomes which mediate genetic variation at a rate greater than ‘natural’ transformation. Mobile genetic elements such as phage and integrative and conjugative elements (ICE) promote more rapid genome evolution in response to strong selection pressure, such as the use of antibiotics in the human host. The ICE in *H. influenzae* is responsible for significant spread of antibiotic resistance in the bacterium and is able to cross the barrier to other species, such as *H. parainfluenzae*[22], at a rate which is greater than that predicted to be achievable through transformation.

## Conclusions

The pair-wise alignment of whole genomes, using Mauve, provided us a useful means to inform on relationships between strains that are influenced by frequent recombination. Our findings provide evidence that transformation-mediated homologous recombination plays a major role in shaping the diversity of natural *H. influenzae* populations and that individual strains contribute to and can acquire genes from the superset of all genes of the species.

## Methods

### Bacterial isolates

Strains were selected for genome sequencing (Table 1) from a collection of more than 1900 strains archived in Oxford. A majority of the strains has been characterised by one or more methods including MLST, MLEE, 16S rRNA sequencing, biotyping, and capsular type. Data on the association of strains with different diseases, dates and geographical sites of isolation were also available for many strains. 46 *H. influenzae* strains were selected for study that represented the diversity within a tree created from the concatenated sequence data from the entire MLST database ( http://haemophilus.mlst.net). A further 15 strains were selected based on existing MLEE and biotype data. Finally, clinical, geographical and temporal data were used to identify some further strains that were included, based on criteria other than MLST or MLEE, as well as a number of strains from closely related species and sub-species of *H. influenzae* including *H. haemolyticus*, *Haemophilus parahaemolyticus, Haemophilus parainfluenzae*, *Haemophilus paraphrophilus*, *H. influenzae* biotype IV strains, and putative ‘hybrid’ *H. influenzae-H. parainfluenzae* strains (Table 1). The latter ‘hybrid’ strains are *H. influenzae* isolates that do not contain a *fucK* MLST allele, a characteristic of *H. parainfluenzae*, and therefore their classification is uncertain (personal communication Abdel Elamin, University of Oxford). Most of the serotype b strains were recovered from patients with invasive disease but a number were associated with non-symptomatic carriage.

Bacterial isolates were cultured from frozen on solid brain heart infusion (BHI) medium supplemented with 10% Levinthals reagent and 1% agar, and incubated at 37°C. For DNA preparation, bacteria were cultured on BHI liquid supplemented with haemin (10 μg/ml) and NAD (2 μg/ml).

### Genome sequencing, assembly, and comparison of genome sequence data

Strains were grown on BHI broth and chromosomal DNA was isolated from bacteria using Qiagen columns as described by the supplier. The genomic DNA from 96 strains was sequenced using multiplex (12 separately indexed DNAs per lane) Illumina sequencing as described previously [21]. The sequencing was conducted utilising 7 lanes (84 DNAs) on one flow cell and one lane (12 DNAs) on a second flow cell. The 55 bp reads from each of the 96 strains were separated using the index tags, and then assembled using the Velvet assembly programme [14]. Genome sequences for eleven strains were rejected due to poor assembly; the result of insufficient coverage or large numbers of small contigs (lower part of Table 1). For 85 *Haemophilus* strains, genome sequences of between 1.27 Mbp to 1.91 Mbp in length were assembled by Velvet (Table 1).

The sequence reads were mapped to a reference using MAQ [15] and default parameters, these were then tested to identify the depth of reads covering the lower %G+C regions of DNA, as an indication of when coverage was insufficient for assembly.

A SNP-based tree was generated by mapping the Illumina fastq sequences against the reference sequence of Hib strain 101810 (acc. number FQ312006) using SMALT version 0.6.3 software, SNPs were called and a tree generated from the SNP alignment using FastTree.

### Serotyping

The serotype of predicted type b strains was determined by the slide agglutination test using serotype-specific serum as described elsewhere [23]. The results from these tests were supported by BLAST analysis of the respective genome sequence derived in this study using published type b capsule gene sequence as a probe.

### Transformation of *H. influenzae*

Genomic DNAs from strains Eagan and a spontaneous high level streptomycin resistant derivative, Eaganstr^R^, were prepared and then used to transform strain Rd using the standard MIV protocol [24]. Transformants were selected following growth overnight on BHI plates with or without added streptomycin (500 μg/ml). 200 independent colonies were selected, pooled, and genomic DNA was isolated from the respective Rd+Eaganstr^R^ and Rd+Eagan transformants. The pooled genomic DNA from each transformation was sequenced on an individual Illumina GAII flow cell at the Wellcome Trust Sanger Institute. The frequency of spontaneous str^R^ mutation was calculated by plating on BHI/streptomycin plates competent Rd cells taken through the transformation procedure but without added donor DNA.

## Abbreviations

Hib: Serotype b *Haemophilus influenzae*; LPS: Lipopolysaccharide; MLEE: Multi-locus enzyme electrophoresis; MLST: Multi-locus sequence typing; NTHi: Non-typeable *Haemophilus influenzae*; SNP: Single nucleotide polymorphism.

## Competing interests

The authors have no competing interests.

## Authors’ contributions

PP, ERM and DWH designed the study and PP carried out the analyses of the whole genome sequence data thus obtained. SB and JP facilitated the sequencing of the bacterial genomes. PP, ERM and DWH were the main contributors to the writing of the manuscript, all authors read and approved the final draft.

## Supplementary Material

Additional file 1 **Figure S1.** Tree indicating the relatedness of *Haemophilus *genome sequences based on similarities in their patterns of SNPs. Illumina fastq sequences were mapped against the reference sequence of Hib strain 10810 and the tree was generated using FastTree from the SNP alignments. Some minor differences in strain placement when compared to Mauve analysis reflects those strains with the lowest quantity (and quality) of genome sequence information. Click here for file

## References

[B1] Boissy R Ahmed A Janto B Earl J Hall BG Hogg JS Pusch GD Hiller LN Powell E Hayes J Comparative supragenomic analyses among the pathogens Staphylococcus aureus, Streptococcus pneumoniae, and Haemophilus influenzae using a modification of the finite supragenome model BMC Genomics 12 187 21489287 10.1186/1471-2164-12-187 PMC3094309

[B2] Medini D Donati C Tettelin H Masignani V Rappuoli R The microbial pan-genome Curr Opin Genet Dev 2005 15 6 589 594 10.1016/j.gde.2005.09.006 16185861

[B3] Hogg J Hu F Janto B Boissy R Hayes J Keefe R Post J Ehrlich G Characterization and modeling of the Haemophilus influenzae core and supragenomes based on the complete genomic sequences of Rd and 12 clinical nontypeable strains Genome Biol 2007 8 6 R103 10.1186/gb-2007-8-6-r103 17550610 PMC2394751

[B4] Fleischmann RD Adams MD White O Clayton RA Kirkness EF Kerlavage AR Bult CJ Tomb JF Dougherty BA Merrick JM Whole-genome random sequencing and assembly of Haemophilus influenzae Rd Science 1995 269 5223 496 512 10.1126/science.7542800 7542800

[B5] Kilian M A taxonomic study of the genus Haemophilus, with the proposal of a new species J Gen Microbiol 1976 93 1 9 62 10.1099/00221287-93-1-9 772168

[B6] Musser JM Kroll JS Moxon ER Selander RK Clonal population structure of encapsulated Haemophilus influenzae Infect Immun 1988 56 8 1837 1845 2899551 10.1128/iai.56.8.1837-1845.1988 PMC259491

[B7] Barenkamp SJ Munson RS Granoff DM Subtyping isolates of Haemophilus influenzae type b by outer-membrane protein profiles J Infect Dis 1981 143 5 668 676 10.1093/infdis/143.5.668 6972422

[B8] Barenkamp SJ Munson RS Granoff DM Outer membrane protein and biotype analysis of pathogenic nontypable Haemophilus influenzae Infect Immun 1982 36 2 535 540 6979511 10.1128/iai.36.2.535-540.1982 PMC351261

[B9] Sacchi CT Alber D Dull P Mothershed EA Whitney AM Barnett GA Popovic T Mayer LW High level of sequence diversity in the 16S rRNA genes of Haemophilus influenzae isolates is useful for molecular subtyping J Clin Microbiol 2005 43 8 3734 3742 10.1128/JCM.43.8.3734-3742.2005 16081903 PMC1233939

[B10] Loos BG Bernstein JM Dryja DM Murphy TF Dickinson DP Determination of the epidemiology and transmission of nontypable Haemophilus influenzae in children with otitis media by comparison of total genomic DNA restriction fingerprints Infect Immun 1989 57 9 2751 2757 2788138 10.1128/iai.57.9.2751-2757.1989 PMC313521

[B11] Leaves NI Jordens JZ Development of a ribotyping scheme forHaemophilus influenzae type b European Journal of Clinical Microbiology & Infectious 1994 13 12 1038 1045 10.1007/BF02111824 7534231

[B12] Bouchet V Huot H Goldstein R Molecular Genetic Basis of Ribotyping Clin Microbiol Rev 2008 21 2 262 10.1128/CMR.00026-07 18400796 PMC2292578

[B13] Meats E Feil E Stringer S Cody A Goldstein R Kroll J Popovic T Spratt B Characterization of encapsulated and noncapsulated Haemophilus influenzae and determination of phylogenetic relationships by multilocus sequence typing J Clin Microbiol 2003 41 4 1623 1636 10.1128/JCM.41.4.1623-1636.2003 12682154 PMC153921

[B14] Zerbino DR Birney E Velvet: algorithms for de novo short read assembly using de Bruijn graphs Genome Res 2008 18 5 821 829 10.1101/gr.074492.107 18349386 PMC2336801

[B15] Li H Ruan J Durbin R Mapping short DNA sequencing reads and calling variants using mapping quality scores Genome Res 2008 18 11 1851 1858 10.1101/gr.078212.108 18714091 PMC2577856

[B16] Darling AC Mau B Blattner FR Perna NT Mauve: multiple alignment of conserved genomic sequence with rearrangements Genome Res 2004 14 7 1394 1403 10.1101/gr.2289704 15231754 PMC442156

[B17] Mell JC Shumilina S Hall IM Redfield RJ Transformation of natural genetic variation into Haemophilus influenzae genomes PLoS Pathog 2011 7 7 e1002151 10.1371/journal.ppat.1002151 21829353 PMC3145789

[B18] Druley TE Vallania FL Wegner DJ Varley KE Knowles OL Bonds JA Robison SW Doniger SW Hamvas A Cole FS Quantification of rare allelic variants from pooled genomic DNA Nat Methods 2009 6 4 263 265 10.1038/nmeth.1307 19252504 PMC2776647

[B19] Blattner FR Plunkett G 3rd Bloch CA Perna NT Burland V Riley M Collado-Vides J Glasner JD Rode CK Mayhew GF The complete genome sequence of Escherichia coli K-12 Science 1997 277 5331 1453 1462 10.1126/science.277.5331.1453 9278503

[B20] Hayashi K Morooka N Yamamoto Y Fujita K Isono K Choi S Ohtsubo E Baba T Wanner BL Mori H Highly accurate genome sequences of Escherichia coli K-12 strains MG1655 and W3110 Mol Syst Biol 2006 2 2006 0007 16738553 10.1038/msb4100049 PMC1681481

[B21] Croucher NJ Harris SR Fraser C Quail MA Burton J van der Linden M McGee L von Gottberg A Song JH Ko KS Rapid pneumococcal evolution in response to clinical interventions Science 2011 331 6016 430 434 10.1126/science.1198545 21273480 PMC3648787

[B22] Juhas M van der Meer JR Gaillard M Harding RM Hood DW Crook DW Genomic islands: tools of bacterial horizontal gene transfer and evolution FEMS Microbiol Rev 2009 33 2 376 393 10.1111/j.1574-6976.2008.00136.x 19178566 PMC2704930

[B23] Ingram DL Collier AM Pendergrass E King SH Methods for serotyping nasopharyngeal isolates of Haemophilus influenzae: slide agglutination, Quellung reaction, countercurrent immunoelectrophoresis, latex agglutination, and antiserum agar J Clin Microbiol 1979 9 5 570 574 383743 10.1128/jcm.9.5.570-574.1979 PMC275349

[B24] Herriott RM Meyer EM Vogt M Defined nongrowth media for stage II development of competence in Haemophilus influenzae J Bacteriol 1970 101 2 517 524 5308771 10.1128/jb.101.2.517-524.1970 PMC284936

