# Supplementary figures and images for: Investigations into genome diversity of Haemophilus influenzae using whole genome sequencing of clinical isolates and laboratory transformants
Source: BMC Microbiol. 2012 Nov 23;12:273. doi: 10.1186/1471-2180-12-273 (PMC3539920; doi:10.1186/1471-2180-12-273)

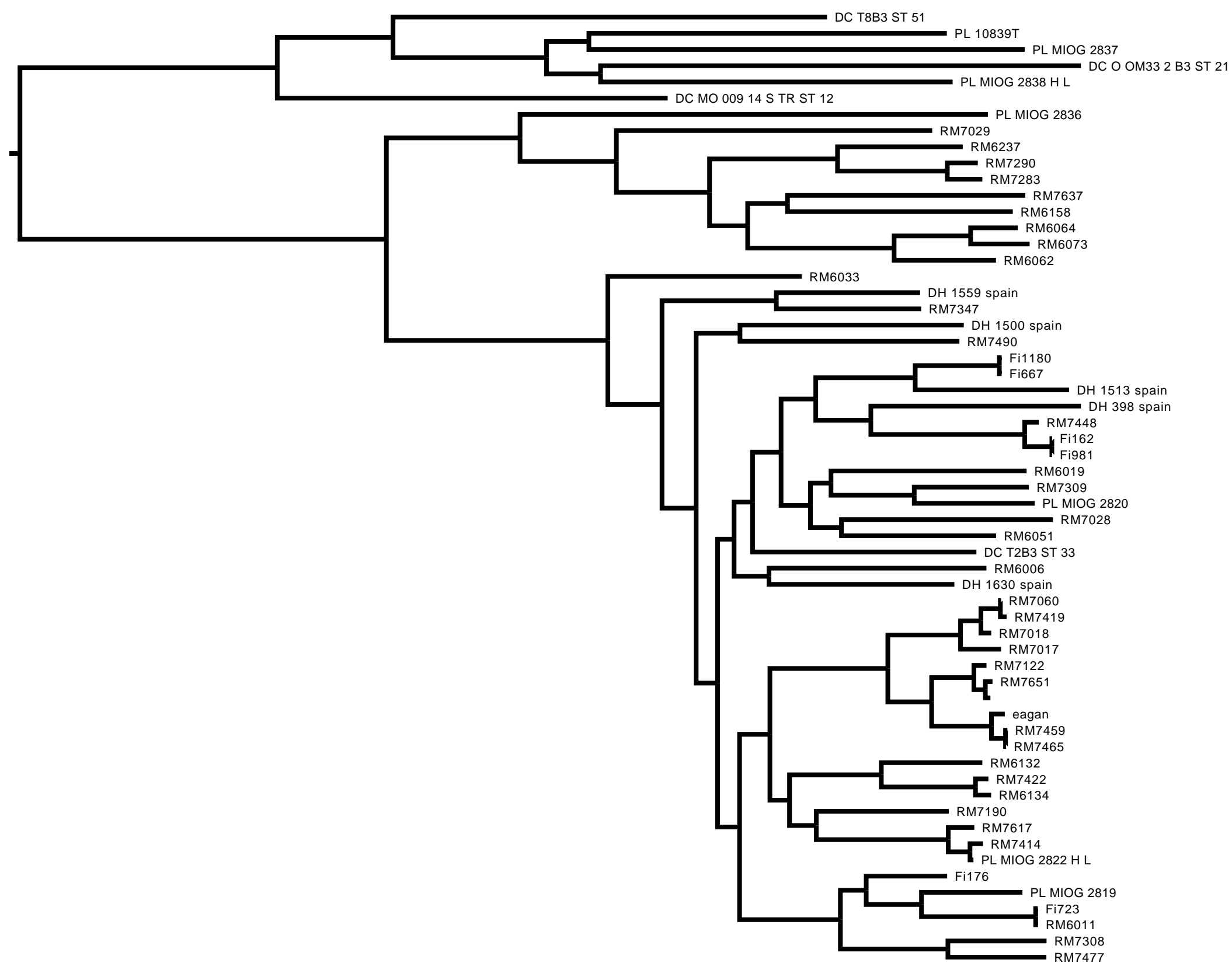

Supplement: Additional file 1 — Figure S1. Tree indicating the relatedness of Haemophilus genome sequences based on similarities in their patterns of SNPs. Illumina fastq sequences were mapped against the reference sequence of Hib strain 10810 and the tree was generated using FastTree from the SNP alignments. Some minor differences in strain placement when compared to Mauve analysis reflects those strains with the lowest quantity (and quality) of genome sequence information. [file 1471-2180-12-273-S1.pdf]
